# Supplementary material for: S3R: Spatially Smooth and Sparse Regression Reveals High-Dimensional Regulatory Networks in Spatial Transcriptomics
Source: bioRxiv. 2025 Sep 11:2025.09.06.674629. Preprint. [Version 1] doi: 10.1101/2025.09.06.674629 (PMC12440005; doi:10.1101/2025.09.06.674629)
Supplement: Supplementary file 1 [file NIHPP2025.09.06.674629v1-supplement-1.pdf]

bioRxiv preprint doi: https://doi.org/10.1101/2025.09.06.674629; this version posted September 11, 2025. The copyright holder for this preprint (which was not certified by peer review) is the author/funder, who has granted bioRxiv a license to display the preprint in perpetuity. It is made available under aCC-BY 4.0 International license.

Supplementary

Evaluation of S3R on simulation data

bioRxiv preprint doi: https://doi.org/10.1101/2025.09.06.674629; this version posted September 11, 2025. The copyright holder for this preprint (which was not certified by peer review) is the author/funder, who has granted bioRxiv a license to display the preprint in perpetuity. It is made available under aCC-BY 4.0 International license.

To rigorously validate the efficacy of the S3R method, we conducted a systematic evaluation using synthetic datasets. Ground-truth coefficient matrices were generated with 3–5 distinct spatial clusters and varying feature sparsity levels to reflect biologically plausible regulatory networks. Additive Gaussian noise (σ=0.5,1,2) was introduced to simulate technical variability, and feature dimensionalities spanned 20, 100, and 1000, representing low to high- dimensional feature spaces. A visual comparison of the clustering accuracy between S3R and four other competing methods, including RSCC, EN, GWL and VS-GPSVC was shown in **Extended Fig. 1a**. In this simulation scenario for the left four columns, the predictor matrix $\boldsymbol{X}$ is of dimension 100×20, depicting a relatively low-dimensional feature setting; the predictor matrix $\boldsymbol{X}$ is of dimension 100×2000 for the right two columns, depicting a high dimensional setting. We introduced different number of coefficient clusters that exist among the response and the predictors, such as 3,4,5,6. The detected clusters by different methods were colored differently. The spatial map on the first row shows the true coefficient matrix. The map in the second row represents the coefficient clusters given by our proposed S3R algorithm, which shows a high degree of similarity with the true spatial dissection, similar for the other methods shown in the rest of the rows.

A more detailed quantitative evaluation is presented in Extended **Fig. 1b**, where we report the Adjusted Rand Index (ARI) between the true coefficient clusters, and the coefficient clusters given by different methods, obtained under five cluster scenarios (3–6 clusters) and three noise levels (σ = 0.5, 1, 2). The first four columns represent the low-dimensional settings (N × p = 100 × 20 to 2000 × 20), while the last two illustrate the high-dimensional cases (N × p = 200 × 1000 and 400 × 1000). Across all panels, ARI scores decline as the Gaussian noise increases, yet S3R consistently attains the highest values, outperforming RSCC, EN, GWL, and VS-GPSVC in every configuration. Notably, the gap between S3R and the competing methods widens in the high-dimensional regime, underscoring the effectiveness of its sparsity and spatial smoothness constraints when p far exceeds N.

Runtime comparison of S3R and competing methods

To assess computational scalability, we compared the runtime of S3R with RSCC, Elastic Net (EN), Graph-guided Weighted Lasso (GWL), and VS-GPSVC across datasets with varying sample sizes (N = 200–2000) and feature dimensions (P = 20–1000). As shown in Supplementary **Fig. 1**, all methods perform similarly in low-dimensional settings (e.g., N = 200, P = 20), although GWL consistently incurs higher runtime. With increasing dimensionality (e.g., N = 400, P = 100; N = 1000, P = 100), VS-GPSVC becomes substantially slower. In high-dimensional regimes (e.g., N = 200–2000, P = 1000), S3R maintains practical runtime while several competing methods either slow down considerably or fail to complete (red “X” marks). Across all scenarios, S3R achieves favorable runtime relative to accuracy. All runtime experiments were conducted on a personal PC.

bioRxiv preprint doi: https://doi.org/10.1101/2025.09.06.674629; this version posted September 11, 2025. The copyright holder for this preprint (which was not certified by peer review) is the author/funder, who has granted bioRxiv a license to display the preprint in perpetuity. It is made available under aCC-BY 4.0 International license.

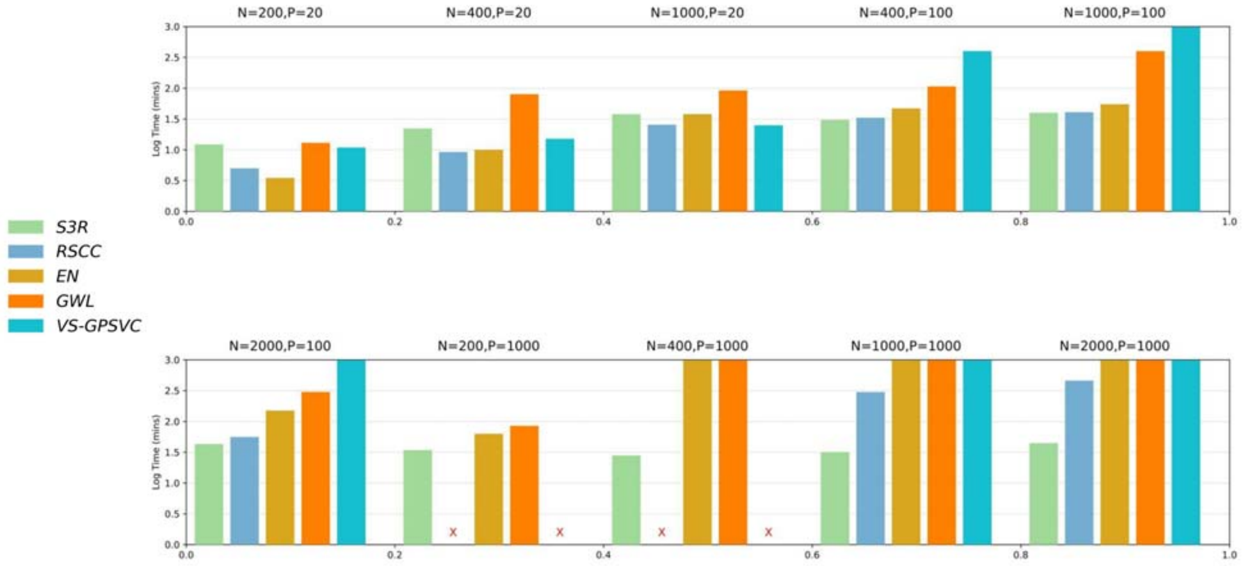

Supplementary Figure 1 | The runtime comparison of S3R and other methods.
